# Prevention of Herpes Simplex Virus Induced Stromal Keratitis by a Glycoprotein B-Specific Monoclonal Antibody

**DOI:** 10.1371/journal.pone.0116800

**Published:** 2015-01-14

**Authors:** Adalbert Krawczyk, Miriam Dirks, Maren Kasper, Anna Buch, Ulf Dittmer, Bernd Giebel, Lena Wildschütz, Martin Busch, Andre Goergens, Karl E. Schneweis, Anna M. Eis-Hübinger, Beate Sodeik, Arnd Heiligenhaus, Michael Roggendorf, Dirk Bauer

**Affiliations:** 1 Institute of Virology, University Hospital Essen, University of Duisburg-Essen, Essen, Germany; 2 Ophtha-Lab, Department of Ophthalmology at St. Franziskus Hospital, Muenster, Germany; 3 Institute of Virology, Hannover Medical School, Hannover, Germany; 4 Institute for Transfusion Medicine, University Hospital Essen, Essen, Germany; 5 Institute of Virology, University Medical Center Bonn, Bonn, Germany; UC Irvine Medical Center, UNITED STATES

## Abstract

The increasing incidence of acyclovir (ACV) and multidrug-resistant strains in patients with corneal HSV-1 infections leading to Herpetic Stromal Keratitis (HSK) is a major health problem in industrialized countries and often results in blindness. To overcome this obstacle, we have previously developed an HSV-gB-specific monoclonal antibody (mAb 2c) that proved to be highly protective in immunodeficient NOD/SCID-mice towards genital infections. In the present study, we examined the effectivity of mAb 2c in preventing the immunopathological disease HSK in the HSK BALB/c mouse model. Therefore, mice were inoculated with HSV-1 strain KOS on the scarified cornea to induce HSK and subsequently either systemically or topically treated with mAb 2c. Systemic treatment was performed by intravenous administration of mAb 2c 24 h prior to infection (pre-exposure prophylaxis) or 24, 40, and 56 hours after infection (post-exposure immunotherapy). Topical treatment was performed by periodical inoculations (5 times per day) of antibody-containing eye drops as control, starting at 24 h post infection. Systemic antibody treatment markedly reduced viral loads at the site of infection and completely protected mice from developing HSK. The administration of the antiviral antibody prior or post infection was equally effective. Topical treatment had no improving effect on the severity of HSK. In conclusion, our data demonstrate that mAb 2c proved to be an excellent drug for the treatment of corneal HSV-infections and for prevention of HSK and blindness. Moreover, the humanized counterpart (mAb hu2c) was equally effective in protecting mice from HSV-induced HSK when compared to the parental mouse antibody. These results warrant the future development of this antibody as a novel approach for the treatment of corneal HSV-infections in humans.

## Introduction

Ocular Herpes Simplex Virus type 1 (HSV-1) induced keratitis is one of the leading causes of infectious blindness in the industrialized world. The global incidence of HSV-induced ocular disease is roughly 1.5 million, including an estimated number of 40.000 new cases of severe monocular visual impairment or blindness each year [1]. HSV-1 infections of the cornea frequently result in disease ranging from mild epithelial inflammations to severe immune mediated chronic ulcerations of the cornea, such as severe necrotizing stromal keratitis, also called Herpetic Stromal Keratitis (HSK) [2, 3]. After primary infection of the cornea the virus replicates in the corneal epithelium and migrates to the trigeminal ganglion by moving directly between adjacent epithelial cells, from epithelial cells to neurons, by intracellular axonal transport and by transfer across neuronal synapses for spread from first order to second-order neurons [4]. Both, the cell-to-cell spread and the intracellular axonal transport are the key mechanisms of HSV to facilitate rapid viral dissemination and to escape from the host cellular and humoral immune defense systems [5]. HSV establishes latent, asymptomatic infections in neurons of the peripheral nervous system. Frequent periodical reactivations of the latent virus and its transmission from the trigeminal ganglia to the periphery through the cell-to-cell spread may lead to recurrent infections of the cornea associated with severe T-cell mediated inflammatory lesions that finally may result in HSK [6] and blindness [7]. Currently, systemic or topical treatment with acyclovir (ACV) is successfully used to suppress the viral replication in patients with recurrent herpes reactivation. Besides, corticosteroids are used to suppress immune responses in the cornea to avoid corneal scarring. Recent studies have shown that the incidence of acyclovir resistant HSV-1 strains has dramatically increased to roughly 6.4% in immunocompetent patients with HSK [1, 8]. Due to multiple serious side effects the use of ganciclovir (GCV) or foscarnet (FOS) is limited [9]. Furthermore, crossresistances towards GCV, FOS or cidofovir (CDV) are increasingly observed [10]. It is therefore essential to develop novel, well-tolerated treatment options for patients with recurrent acyclovir- or cross-resistant HSV-1 infections of the cornea.

In prior studies we have reported that the monoclonal antibody mAb 2c was developed as a highly potent compound for neutralization of drug resistant Herpes Simplex Viruses [11, 12]. This antibody recognizes a common epitope on the glycoprotein B of HSV-1 and HSV-2 and exhibits extraordinarily high antiviral efficacy in vitro and in highly immunodeficient NOD/SCID mice. The epitope sequence recognized by mAb 2c is highly conserved in HSV-1 and HSV-2 isolates and proved to be essential for virulence and viral fitness [13]. In contrast to the vast majority of human antibodies generated during HSV infection or induced by vaccination with a recombinant protein vaccine, this antibody does not only neutralize the circulating virus, but also inhibits the HSV transmission by cell-to-cell spread [11]. This mechanism is known to be crucial during the establishment of latency and also during reactivations [5, 14]. Due to the unique properties of mAb 2c, we designed the humanized derivate mAb hu2c for the treatment of HSV infections resistant to standard antiviral medication [11, 12]. Both the humanized and the murine antibody exhibited equal binding properties and are capable of neutralizing a broad range of clinical isolates resistant to ACV, FOS or CDV. In vivo, mAb hu2c also prevented NOD/SCID mice against lethal HSV-1 infection with a multidrug-resistant clinical isolate [15]. Based on these promising data, we concluded that this antibody should be an appropriate treatment option for severe corneal HSV-1 infections inducing HSK.

Consequently, the objectives of the present study were to examine the neutralizing capacity of the HSV-gB-specific monoclonal antibody towards the eye-pathogenic HSV-1 strain KOS and ACV-resistant clinical isolates in vitro and its efficacy in prevention of HSK in the immunocompetent murine BALB/c model.

## Materials and Methods

### Ethics statement

Animal experiments were performed in strict accordance with the German regulations of the Society for Laboratory Animal Science (GV-SOLAS) and the European Health Law of the Federation of Laboratory Animal Science Associations (FELASA). The protocol was approved by the North Rhine-Westphalia State Agency for Nature, Environment and Consumer Protection (LANUV) (Permit number: G 1194/11). Preparation of murine sensory neurons was performed according to the German Animal Welfare Act. All efforts were made to minimize suffering. For immunofluorescence microscopy experiments (see below), we used human serum of healthy, HSV‑1 seronegative volunteers. Written informed consent of the blood donors was obtained. Permission was granted by the Institution Review Board of the Hannover Medical School (approval number 893).

### Animals

Female BALB/c mice, 8 weeks of age, were purchased from Charles River Laboratories (Charles River Laboratories, Sulzfeld, Germany) and maintained under pathogen free conditions. All in vivo experiments were conducted according to the German legal requirements with the approval of the University Hospital Essen’s animal facility. To isolate primary neurons, C57Bl/6JHanZtm mice were bred and maintained at the Hannover Medical School Laboratory Animal Facility.

### Cells

Vero cells (American Type Culture Collection, ATCC, CCL81, Rockville, MD) were cultured in Dulbecco’s Modified Eagle Medium (DMEM, Life Technologies Gibco, Darmstadt, Germany) containing 10% (v/v) fetal calf serum (FCS; Life Technologies Gibco), 100 U/ml penicillin and 0.1 mg/ml streptomycin. BHK-21 cells (ATCC CCL-10) were cultured in Minimum Essential Medium (MEM, Cytogen, Sinn, Germany) supplemented with 10% FCS (v/v), 100 U/ml penicillin and 0.1 mg/ml streptomycin. C127I epithelial cells (ATCC CRL-1616) were cultured in DMEM (Life Technologies Gibco) containing 10% (v/v) FCS (PAA, Saarbrücken, Germany), 100 U/ml penicillin and 0.1 mg/ml streptomycin. Primary cultures of dorsal root ganglion (DRG) neurons were prepared as described previously [16, 17]. Such neurons are infected in the course of human and murine HSV-1 infections [18–21]. Briefly, adult C57BL/6J mice were sacrificed, DRG from the cervical, thoracic and lumbar level of the animals were dissected and collected in 1x Hank’s Balanced Salt Solution (HBSS, containing 5 mM HEPES, 10 mM D-Glucose, pH 7.4). Ganglia were first digested for 20 min at 37°C with 20 mg/ml papain (Sigma-Aldrich, Schnelldorf, Germany) in papain activation solution (0.4 mg/ml L-Cysteine, 0.5 mM EDTA, 1.5 mM CaCl_2_ × 2H_2_O, pH 7.4) followed by digestion with 10 mg/ml collagenase IV (Invitrogen) and 12 mg/ml dispase II (Sigma-Aldrich) in 1xHBSS. Ganglia were pelleted and resuspended in 1 ml 1xHBSS and triturated using Pasteur pipettes with narrowed ends. The neuron-suspension was spun for 8 min at 381 × g through a cushion consisting of 20% (v/v) Percoll in CO_2_-independent medium (Life Technologies Gibco) containing 10 mM D-glucose, 5 mM HEPES, 10% FCS, 100 U/ml penicillin and 0.1 mg/ml streptomycin. After removing the supernatant, the cell pellet was resuspended in 2 ml CO_2_‑independent medium and finally centrifuged for 2 min at 1000 × g. The pellet was resuspended in Ham’s F-12 nutrient mix medium containing 10% FCS, 50 ng/ml 2.5S NGF (Promega Corporation, Fitchburg, WI, US), 100 U/ml penicillin and 0.1 mg/ml streptomycin and seeded onto cover slips coated with 0.01% (w/v) in H_2_O poly-L-lysine (150,000–300,000 g × mol^-1^ Sigma-Aldrich) and natural murine laminin (0.8 µg per cover slip, Invitrogen) within 24-well plates. The neurons were cultivated at 37°C and 5% CO_2_ in a humidified incubator and medium was changed twice per week. The antimitotic drug 1-β-D-arabinofuranosylcytosine (Sigma-Aldrich) was added to a final concentration of 2 µM to suppress proliferation of dividing, non-neuronal cells and was washed out prior to infection. After one week of cultivation, neurons were used for conducting the experiments.

### Viruses

The eye pathogenic strain HSV-1 strain KOS and ACV-resistant isolates were propagated on Vero cells and stored at -80°C. For examination of virus titers the cell supernatants or organ homogenizates were titrated on Vero cells as previously described [22]. Three clinical isolates with a resistance towards ACV [10] were kindly provided by Georges M.G.M. Verjans (Department of Virology, Erasmus Medical Centre, Rotterdam, the Netherlands). The reporter virus HSV-1(17^+^)Lox-_pMCMV_mCherry, short HSV-1(17^+^)Lox-Che, has been derived from HSV‑1(17^+^)Lox-_pMCMV_GFP [23]. The GFP gene of HSV-1(17^+^)Lox-GFP has been replace by the gene for monomeric Cherry (R. Budida, A. Pohlmann, B. Sodeik, and G. Behrens; to be published elsewhere); both strains expressing the fluorescent proteins GFP or mCherry as a surrogate markers can be used to monitor HSV-1 early viral gene expression. HSV-1(17^+^)Lox-Che was propagated using BHK-21 cells and purified as described previously [24, 25]. The titer was determined by plaque assays, the genome to plaque forming units (PFU) ratio was determined by real time PCR [24, 25].

### Antibodies

Monoclonal antibodies mAb 2c and mAb hu2c were purified from serum-free hybridoma- or SP2/0 cell supernatants by protein A chromatography as described before [11, 15]. Purity was confirmed by FPLC ≥ 95%. Concentration was measured with a NanoDrop 2000 spectrometer.

### Neutralization assay

The neutralizing capacity of mAb 2c was determined on Vero cells by endpoint dilution as previously described [11]. Briefly, serial dilutions of antibodies were incubated with 100 TCID_50_ of HSV-1 KOS or an ACV-resistant clinical isolate for 1 h at 37°C in cell culture medium. The antibody virus inoculum was applied to Vero cell monolayers grown in 96-well plates, and the cytopathic effects (CPEs) were scored after 48 h of incubation at 37°C. The antibody concentration required for complete inhibition of virus-induced CPE was determined as the neutralization titer. [11, 15].

### Inhibition of the cell-to-cell spread

The effectivity of mAb 2c to inhibit cell-to-cell transmission of the eye pathogenic strain HSV-1 KOS was analyzed by immunofluorescence as previously described [11]. Briefly, confluent monolayers of Vero cells, grown in 24-well tissue culture plates, were infected with 100 TCID_50_ of HSV-1 KOS. After 4 h of adsorption at 37°C the viral inoculum was removed. Cells were incubated for 48 h in DMEM containing 2% FCS in the presence of 500 nM (75µg/ml) mAb 2c, pooled human sera (1:40 diluted in medium) derived from donors with high titers of anti-HSV-immunoglobulins (neutralizing titer 1:256 for total neutralization of 100 TCID_50_ of HSV-1 KOS), or medium alone. The antibody concentrations in cell supernatants represent excess concentrations required for the complete neutralization of the secreted virus. Plaque formation was detected by immunofluorescence. HSV-1 infected cells were stained with a mouse anti-HSV-1/2-gD-antibody (Acris Antibodies, San Diego, CA, USA) and an Alexa Fluor 488 goat anti-mouse IgG specific secondary antibody (Invitrogen). Bound human or murine antibodies were stained with Cy3-conjugated goat anti-mouse IgG or goat anti-human IgG secondary antibodies (Invitrogen), respectively. Immunofluorescence images were acquired with a Zeiss Observer Z1 fluorescence microscope (Carl Zeiss, Oberkochen, Germany) at a 100-fold magnification.

### Analysis of the epithelial cell-to-neuron and neuron-to-epithelial cell spread

Naïve C127I cells were transfected with the GFP expressing plasmid pEGFP-N1 (Invitrogen) for identification. Transfection was performed with the GeneJuice reagent according to the manufacturer’s protocol (Merck Millipore, Darmstadt, Germany). Cells were reversely transfected and incubated for 24 h prior to use. To test the effect of different antibodies on HSV-1 neuron-to-epithelial cell transmission, DRG neurons were infected with 2.5x10^7^ PFU/ml of HSV-1(17^+^)Lox-Che. At 24 h post infection, transfected C127I cells were detached with accutase (GE Healthcare Europe, Freiburg, Germany) and mixed with the antibodies hu2c, 2c or with pooled human IgGs (Anti-HSV-positive; Sigma-Aldrich) at 75 µg/ml or mock treated. Such cell-antibody mixtures were then seeded on top of the infected DRG neurons. To examine the impact of mAbs 2c and hu2c on the epithelial cell-to-neuron spread, C127I cells were infected with 7.5 × 10^6^ PFU/ml for 15 hours. Cells were then detached with accutase, mixed with the indicated antibodies as described above, and seeded on top of naïve DRG neurons. The co-cultured cells were fixed at the indicated time points with PHEMO-fix (68 mM PIPES, 25 mM HEPES, 15 mM EGTA, 3 mM MgCl_2_, 10% (v/v) DMSO, 3.7% (w/v) paraformaldehyde (PFA), 0.05% (v/v) glutaraldehyde, 0.5% (v/v) Triton X-100, pH 6.9) at room temperature for 10 min, and then washed for two times 5 min with PHEMO-buffer (68 mM PIPES, 25 mM HEPES, 15 mM EGTA, 3 mM MgCl_2_, 10% (v/v) DMSO, pH 6.9) for 5 min at 37°C [26, 27]. Residual fixative was quenched with 50 mM NH_4_Cl in PBS. Unspecific binding sites and the Fc-receptor of the gE/gI complex of HSV-1 [28] were disarmed by incubation with blocking reagent containing 5% (w/v) BSA and 10% (v/v) human HSV-1 seronegative serum in PBS for 30 min. Antibodies were diluted in blocking reagent and incubated with the cells in a humidified chamber for 30 to 60 min. The monoclonal antibody directed against β-tubulin-III (mouse mab5564, Merck Millipore, Darmstadt, Germany) was used to identify neurons. Secondary antibodies for immunofluorescence microscopy against mouse were conjugated to Alexa Fluor (Life Technologies Gibco). Finally, samples were embedded in mounting medium (6 g glycerol, 2.6 g Mowiol 40–88, 6 ml H_2_O, 12 ml 0.2 M Tris, pH 8.5) containing 0.1 g/ml DABCO (1,4-Diazabicyclo[2, 2, 2]octane). Samples were analyzed with a Zeiss Axiovert 200M microscope equipped with a LSM 510 Meta confocal laser-scanning unit with argon (Argon2, 488 nm) and helium-neon (HeNe1, 543 nm, HeNe1, 633 nm) lasers using a plan-apochromatic 63x oil-immersion objective with a numeric aperture of 1.4. Images were acquired with the Zeiss LSM Image Browser (version 4.2.0.121), analyzed with ImageJ (Version 1.45 h, Wayne Rasband, National Institute of Health, USA, http://rsb.info.nih.gov/ij/) and processed with Adobe Photoshop CS4 (Version 11.0, Adobe Systems Inc., San Jose, CA, USA). A circular area of 85 µm^2^ was placed into the center of a neuron, as identified by the β-tubulin-III-labeling or a transfected C127I cell, as identified by the GFP expression.

### Corneal infection of mice and study design

Mice were anesthetized by intraperitoneal injection of ketamine hydrochloride (2 mg) and mepivacaine hydrochloride (400 ng). The epithelium of the right eye was scratched eight times in a criss-cross pattern and inoculated with 1 × 10^5^ PFU of HSV-1 KOS in 5 µl medium [29]. Infected mice were either systemically or topically treated with the murine antibody mAb 2c. Topical treatment was carried out by periodical inoculations (5 times per day) of the infected eye with 5 µl (28.5 µg) antibody solution (eye drops) starting at 24 h post infection (p.i.) till day 7 p.i.. Systemic treatment was carried out by intravenous administration of 300 µg mAb 2c or mAb hu2c 24 h prior to infection for pre-exposure prophylaxis, or 24, 40, and 56 hours after infection for postexposure immunotherapy. The course of disease was characterized by determination of the severity of the disease (clinical score of blepharitis, epithelial defects and HSK) with an operation microscope (Zeiss, Oberkochen, Germany), each on a scale of 0 to 4, being consistent with inflammation of the eye lids, cytopathic effects of the corneal epithelial cells or corneal opacity with neovascularization, edema and necrosis [22]. Viral loads of infected eyes were measured on day 5 post infection with a standard plaque assay (N = 6 in each group) [30]. The inflammatory cells in the corneas were counted as previously described [31]. Total cell numbers in spleens and lymph nodes were counted after the homogenization of the organs with a 70 µm cell strainer (BD Biosciences, Franklin Lakes, NJ, USA).

### Cytokine quantification by ELISA

Draining lymph nodes (DLN) and spleens from HSV-1 KOS infected mice receiving either antibody treatment or PBS were collected on day 14 after infection. Afterwards the organs were homogenized and 5 × 10^6^ cells were cultivated in triplicates in the presence of 2 × 10^7^ PFU UV-inactivated HSV-1 KOS or medium alone. After 24 hours, the amounts of IL-2 and interferon (IFN)-γ in cell supernatants were quantified by ELISA (OptEIA, Pharmingen, Hamburg, Germany) as previously described [32].

### Proliferation assay

Antigen- and mitogen-induced proliferation from splenocytes or cells of draining lymph nodes was assessed via a flow-cytometry based assay as previously described [33]. Briefly, lymphocytes were stained with eFluor 670 (eBioscience, Frankfurt am Main, Germany) according to the manufacturers instructions. 1 × 10^5^ cells/well were cultured in a 96-well round bottom plate with media, UV-HSV-1 (2 × 10^7^ PFU/ml before UV-inactivation) or Concanavalin A (Biochrom, Berlin, Germany). Due to cell division, cells lose half of the fluorescence with each cell division. After 4 days, cells were surface stained according manufacturers instructions (eBioscience) using the following monoclonal antibodies: rat-anti mouse-CD4-PE, rat-anti-mouse-CD8-FITC, rat IgG2a k isotype control FITC, and rat IgG2b K isotype control PE. To distinguish between viable and non-viable cells, cells were stained with 7-AAD according to manufacturers instructions (BD, Heidelberg, Germany). Proliferation was assessed via flow-cytometry (FACScalibur, BD). At least 2000 viable 7-AAD negative (BD) CD4^+^ (eBioscience, Germany) and 1000 CD8^+^ (eBioscience) were included for analysis. Flow-cytometric data were analyzed via Cytomation Summit Offline V3.1. software (Dako, Hamburg, Germany).

### Trigeminal ganglion reactivation assay

For detection of latent virus, trigeminal ganglia (TG) were explanted on day 14 after infection and co-cultivated with Vero cell monolayers for three weeks as previously described [34]. For the detection of cytopathic effects cultures were examined at daily intervals.

### DNA quantification

HSV-1 genomes were quantified by real-time PCR as previously described [11]. Briefly, DNA was purified from trigeminal ganglia of HSV-1 KOS infected mice using the MagNA Pure LC automated nucleic acid extraction system (Roche, Penzberg, Germany) according to the manufacturer’s instructions. Total virus DNA was then quantified by real‑time PCR (LightCycler; Roche) using the artus HSV-1/2 LC PCR Kit (Qiagen, Hilden, Germany). The analytical detection limit for HSV-1-DNA isolated from trigeminal ganglia was determined to be 200 copies/ganglion according to the manufacturer’s protocol.

### Histological staining

For light microscopy analysis, eyes were fixed (64% isopropanol, 3.7% formaldehyde, 2.5% acetic acid), dehydrated with isopropanol, and embedded in paraffin as previously described [35]. Five-micrometer sections were then stained with hematoxylin-eosin and analyzed by light microscopy.

### Detection of HSV-specific antibodies in sera and tear fluids

Sera (60 µl) and tear fluids (10 µl fluid derived from ocular rinsing with PBS) from HSV-1 KOS infected mice were harvested on day 14 post infection and examined for binding towards HSV‑1 infected Vero cells by flow cytometry as described previously [11]. Data were analyzed using Flowjo version 7.2.5 (Tree Star Inc., Ashland, OR, USA).

### Detection of systemically applied humanized antibody in HSV-1 KOS infected cornea

To investigate whether systemically applied antibodies may reach the infected cornea, we examined corneal tissue sections from HSV-1 KOS infected mice after intravenous injection of mAb hu2c by immunofluorescence. Therefore, mice were corneally infected as described above and a single dose of 300 µg of the humanized antibody mAb hu2c was intravenously injected 48 h post infection. Control mice received PBS. Six hours later the eyes were removed and frozen in liquid nitrogen. Frozen eyes were embedded in Tissue-Tek O.C.T. medium (Sakura, Alphen aan den Rijn, The Nederlands) and sectioned (7 µm) with the Frigocut 2800 microtome (Reichert-Jung, Nussloch, Germany). Frozen sections were dried for 30 min, fixed with cooled acetone for 10 min and incubated with blocking buffer (10% FCS in PBS) for 15 minutes. Subsequently, the corneal sections were stained simultaneously for HSV-1 infection and bound mAb hu2c with polyclonal goat anti-HSV-1 FITC conjugated antibodies (Bethyl laboratories, Montgomery, USA) and a Cy3-conjugated goat anti-human IgG secondary antibody (Invitrogen, Darmstadt, Germany). Nuclei were stained with Hoechst (Hoechst 33342, 1 µg/ml; Sigma, St. Louis, MO) for 5 min according to manufacturer´s protocol. Immunofluorescence images were acquired with a Zeiss Observer Z1 fluorescence microscope at a 200-fold magnification.

### Statistic analysis

Data were analyzed using GraphPadPrism 5 (GraphPad Prism Software, La Jolla, CA, USA). Statistical analysis was performed with nonparametric ANOVA (Kruskal–Wallis) and post hoc Dunn’s multiple-comparisons test or parametric ANOVA (one way analysis) and post hoc Tukey´s multiple-comparisons test. The differences between the number of latently infected trigeminal ganglions and the number of trigeminal ganglions exhibiting reactivation were examined by Fisher’s exact test. Comparisons were considered significant at *P* < 0.05.

## Results

### Effective neutralization of HSV-1 KOS and ACV-resistant clinical isolates

**Figure 1:**
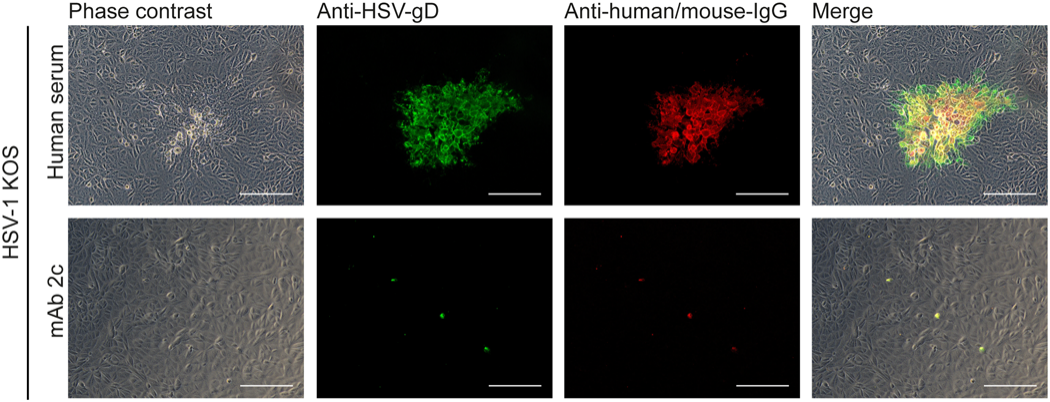
MAb 2c effectively blocks cell-to-cell spread of HSV-1 KOS. Confluent Vero cells were infected with 100 TCID_50_ of HSV-1 KOS and treated with either pooled human polyclonal HSV-neutralizing sera (1:40 in medium) or mAb 2c (500 nM). Cells were stained 48 h after infection for viral transmission with an HSV-1/2-glycoprotein D specific murine antibody and an Alexa488-conjugated secondary anti-mouse or anti-human antibody. Bound human antibodies or mAb 2c were detected with a Cy3-conjugated secondary antibody. Uninfected cells served as negative controls and showed no background staining (not shown). Magnification: 100x. Scale bar: 100 μm.

Cell-to-cell spread is crucial for virus propagation in the skin and mucous membranes as well as for the transmission from epithelial cells to neurons prior to retrograde transport to the trigeminal and dorsal root ganglia, and also for transmission from neurons to epithelial cells after reactivation and anterograde transport. We therefore characterized the antiviral efficacy of the HSV-gB specific monoclonal antibody mAb 2c towards HSV-1 KOS and three acyclovir resistant clinical isolates obtained from patients with HSK [10]. MAb 2c completely neutralized equal viral loads of 100 TCID_50_ of the tested viruses at concentrations of 7.8 nM or 15.6 nM (data not shown). This corresponds to the neutralization efficiency that mAb 2c exhibited to other drug sensitive HSV-1 strains and drug resistant isolates obtained from bone marrow transplants [11]. Like other HSV strains, eye pathogenic HSV-1 strains disseminate by cell-to-cell spread evading the host immune defense [5]. We therefore analyzed the effect of mAb 2c on the cell-to-cell spread of HSV-1 KOS. In contrast to human immunoglobulins (Fig. 1, upper row), mAb 2c limited the infection of the virus to the initially infected cells (Fig. 1, bottom row). Our results show that 500 nM (75 µg/ml) of the mAb 2c was sufficient for complete inhibition of the cell-to-cell spread. These results are consistent with our previous studies with HSV-1 F [11].

**Figure 2:**
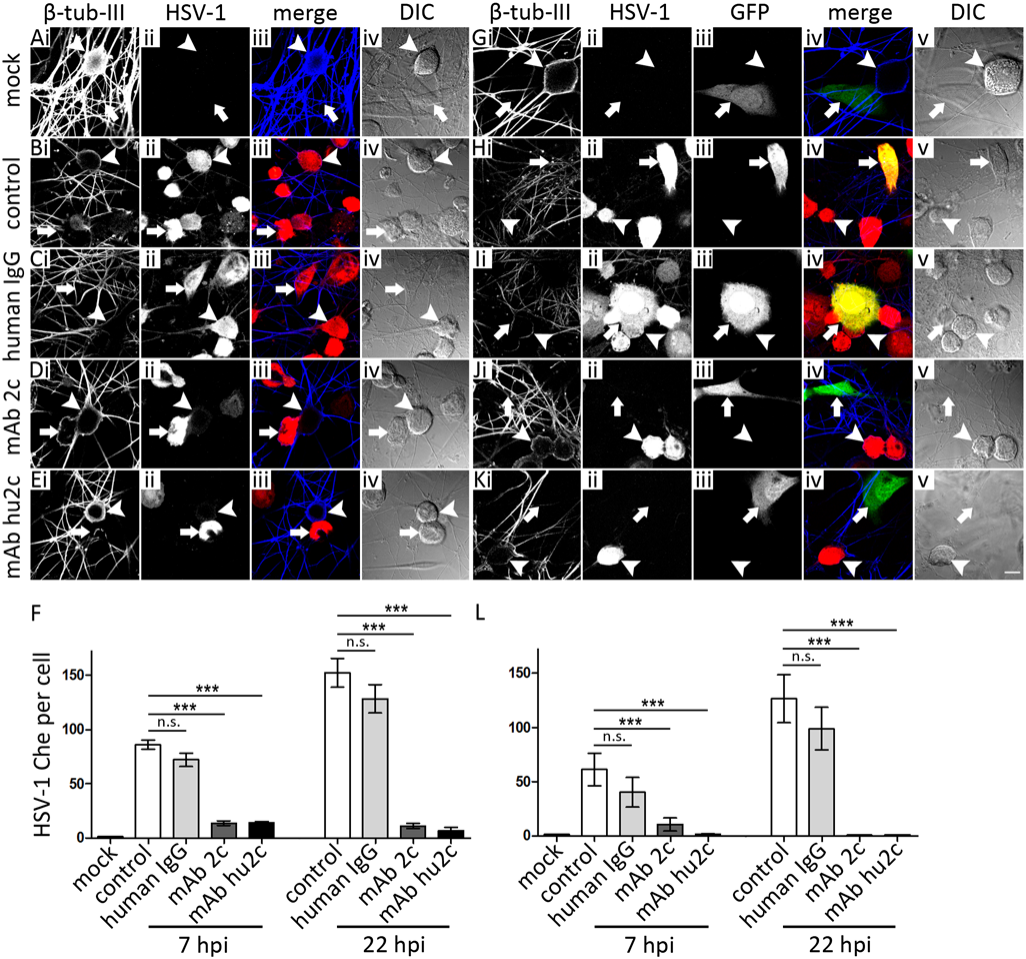
MAbs 2c and hu2c restrict cell-to-neuron **(A-F)** and neuron-to-cell spread of HSV-1 **(G-L)**. For the investigation of cell-to-neuron spread, epithelial C127I cells (arrows) were infected with the reporter virus HSV-1(17^+^)Lox-Che. At 14 hpi cells were detached, treated with the indicated antibodies and co-cultured with uninfected DRG sensory neurons (arrowheads) **(A-E)**. For the investigation of neuron-to-cell transmission, DRG neurons were infected with HSV-1(17^+^)Lox-Che. At 24 hpi, GFP-transfected, uninfected C127I were detached, treated with the indicated antibodies and co-cultured with the infected neurons **(G-K)**. For both settings, cells were fixed as indicated at 7, 22 and 30 hpi with PHEMO and labeled with anti-β-tubulin-III. Images were acquired with a confocal microscope. For the quantification of the cell-to-neuron **(F)** or neuron-to-cell transmission **(L)**, the values of mCherry-fluorescence were determined in a defined area within neurons **(F)** or GFP-positive C127I cells (G). (A-E and G-K) Scale bar is 10 µm. **(F and L)**. Error bars show standard error of the mean (SEM) of one representative experiment. Differences between the groups were statistically significant by a nonparametric ANOVA one way test (***P < 0.001).

To determine whether mAbs 2c and hu2c are capable of inhibiting the direct route of transmission between epithelial and neurons, either uninfected neurons were co-cultured with infected epithelial cells (epithelial cell-neuron spread) or infected neurons were co-cultured with uninfected epithelial cells (neuron-to-epithelial cell spread) in the presence or absence of the indicated antibodies or pooled human IgG as control. To examine the inhibition of cell-neuron spread, dissociated murine DRG were overlaid with HSV‑1(17^+^)Lox-Che infected C127I cells that had been either treated with pooled human IgG (Fig. 2C), mAb 2c (Fig. 2D), humanized mAb 2c (Fig. 2E) or were mock treated (Fig. 2B) as control. The quantification of the cell-to-cell spread is shown as the total fluorescence level induced by HSV-infection (Fig. 2F). For the investigation of the inhibition of the neuron-to-cell spread by mAbs 2c and hu2c the opposite setup was used (Fig. 2G-L). Thus, DRG neurons were infected with HSV‑1(17^+^)Lox-Che and overlaid with GFP-transfected C127I cells that were treated with the indicated antibodies. While the HSV-1 was transmitted from infected C127I cells (arrows in Fig. 2) to neurons (arrowheads in Fig. 2) in the absence of antibodies (Fig. 2B) or in the presence of pooled human IgGs (Fig. 2C), the addition of mAb 2c (Fig. 2Dii) or the humanized mAb hu2c (Fig. 2Eii) entirely inhibited the cell-neuron spread as indicated by lack of primary neurons expressing mCherry. Similarly, the cell-to-cell spread from infected neurons to C127I cells marked by GFP expression was completely inhibited by mAb 2c (Fig. 2Jiv) or hu2c (Fig. 2Kiv). Human IgGs as control had no impact on virus transmission in these experiments (Fig. 2Iiv). Taken together our results demonstrate that mAb 2c mediates effective virus neutralization and inhibition of cell-to-cell transmission of eye-pathogenic HSV-1 strains. Furthermore, mAb 2c as well as the humanized antibody mAb hu2c were capable of inhibiting the HSV transmission between cells and primary neurons.

### Prevention of corneal diseases in mice by systemic antibody application

**Figure 3:**
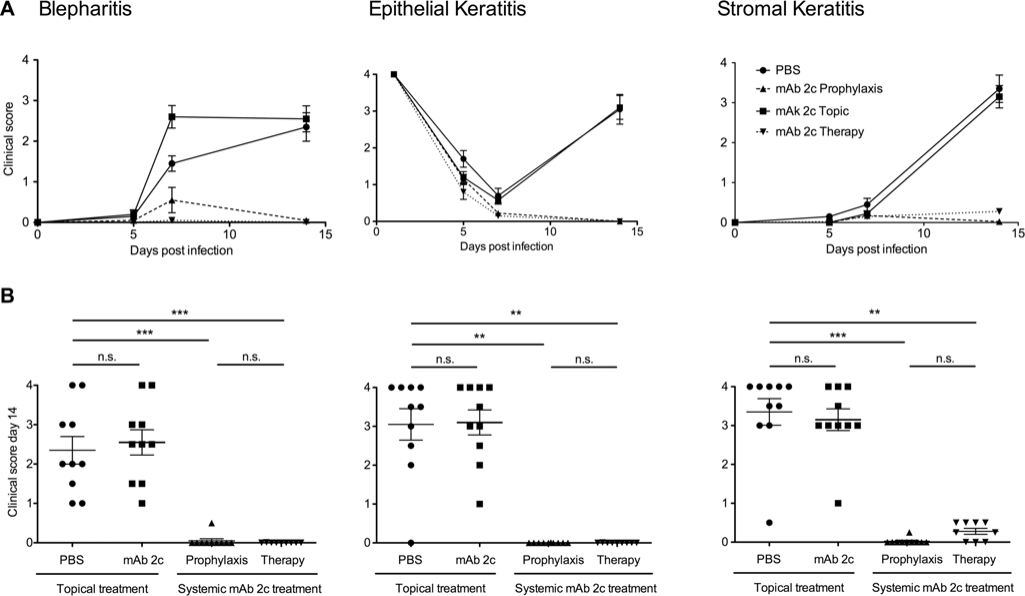
Effective prevention of corneal HSV-1 KOS infection in mice by systemic mAb 2c administration. Ocular disease scores of infected mice for blepharitis, epithelial defects and corneal opacity (stromal keratitis) are shown as an average score of ten mice per group over a period of 14 days **(A)**. The clinical disease scores taken on day 14 after infection are shown as dot plot **(B)**. Every dot represents a single mouse. Differences between the groups were statistically significant by a nonparametric ANOVA one way test (**P < 0.01; ***P < 0.001). Error bars represent the SEM.

**Figure 4:**
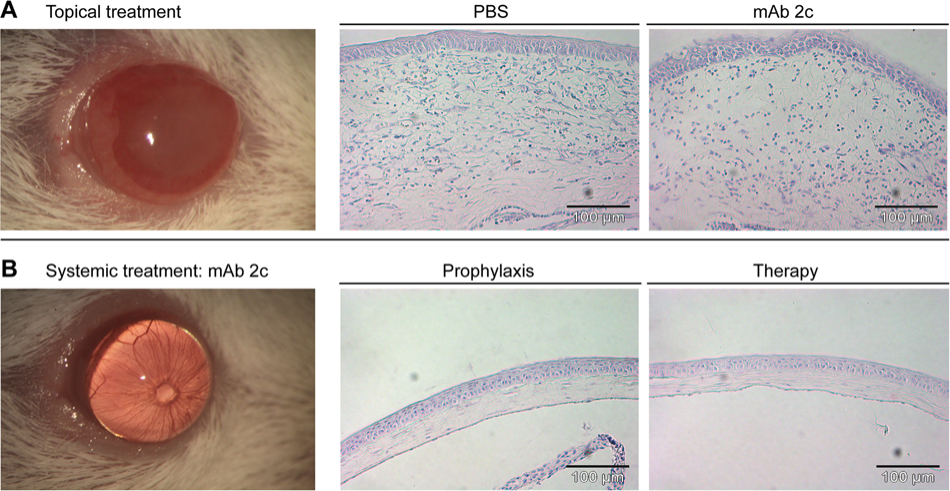
Potent reduction of the severity of HSV-mediated corneal damage by mAb 2c. Groups of ten mice were either topically or systemically treated with mAb 2c. Pictures representative for topically PBS-treated mice **(A)** or systemically treated mice **(B)** were taken at day 14 post infection. Pathological changes of the cornea are shown as representative corneal sections from each group. Hematoxylin-eosin staining. Magnification: 120x. Scale bars: 100 μm.

**Figure 5:**
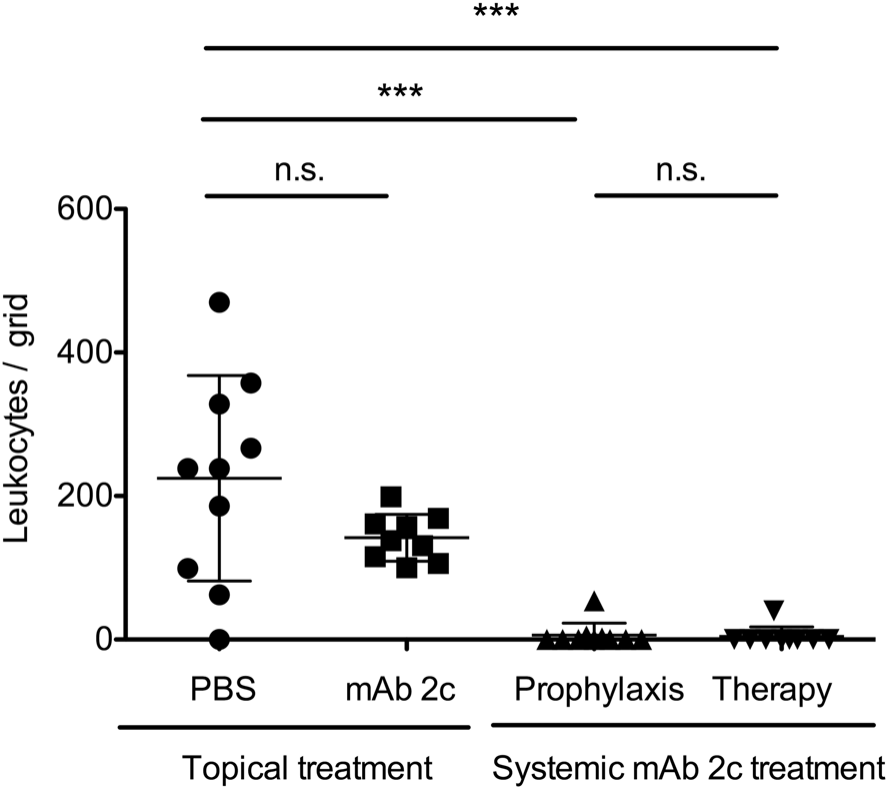
Systemic mAb 2c treatment significantly reduces the leukocyte infiltration of the cornea. Histologically stained corneal sections from each group (seeFig. 3) were examined for the total number of leukocytes infiltrating the stroma. Each dot represents the total cell number in stroma sections of a single mouse. Differences between the groups were analyzed by a nonparametric ANOVA one way test (***P < 0.0001). Error bars represent the SEM.

Based on the unique properties of this antibody, we investigated the antiviral efficacy of topical or systemic mAb 2c treatment on the course of disease in BALB/c mice corneally infected with 1 × 10^5^ PFU of HSV-1 KOS. The antibody was applied topically or systemically. Fourteen days after infection, nine out of ten corneas of the control group showed pronounced HSK with severe necrosis and ulceration, accompanied by highly swollen and inflamed eyelids (Figs. 3 and 4A). The corneal epithelium of these eyes showed epithelial lesions, reflecting viral cytolysis in the early stage, and tissue damage and ulceration in the late stage of disease (Figs. 3 and 4A), while the eye-blink reflex could no longer be induced. Accordingly, an increased number of inflammatory cells, mainly polymorphonuclear (PMN) and mononuclear cells, were found in the central cornea (Fig. 5). The median number of inflammatory cells infiltrating the central cornea in the control group was c = 238.5/grid (Fig. 5). In contrast, the development of HSV-mediated corneal disease was entirely abrogated by systemic antibody application when applied as prophylactic or as post-exposure treatment. The clinical score of blepharitis, epithelial defects and HSK was significantly (P < 0.05) decreased (Fig. 3). All systemically treated mice showed normal eyelids with almost no inflammation. Accordingly, infiltration of the cornea by inflammatory cells was not observed (c = 0/grid; Fig. 4 and Fig. 5), and the eye-blink reflex was intact, similar to uninfected corneas (data not shown). Of note, topical application of mAb 2c had no beneficial effect on the progression of corneal disease (Fig. 3). No significant difference in the degree of cell infiltration between the control group (c = 238.5/grid) and the topically treated group (c = 137.7/grid) was found (Fig. 5). In conclusion, these results demonstrate the successful prevention of HSV-mediated corneal disease by systemic mAb 2c application. There is also strong evidence that the topically applied antibody had no therapeutic effect.

### Influence of mAb 2c treatment on anti-HSV immune reaction

**Figure 6:**
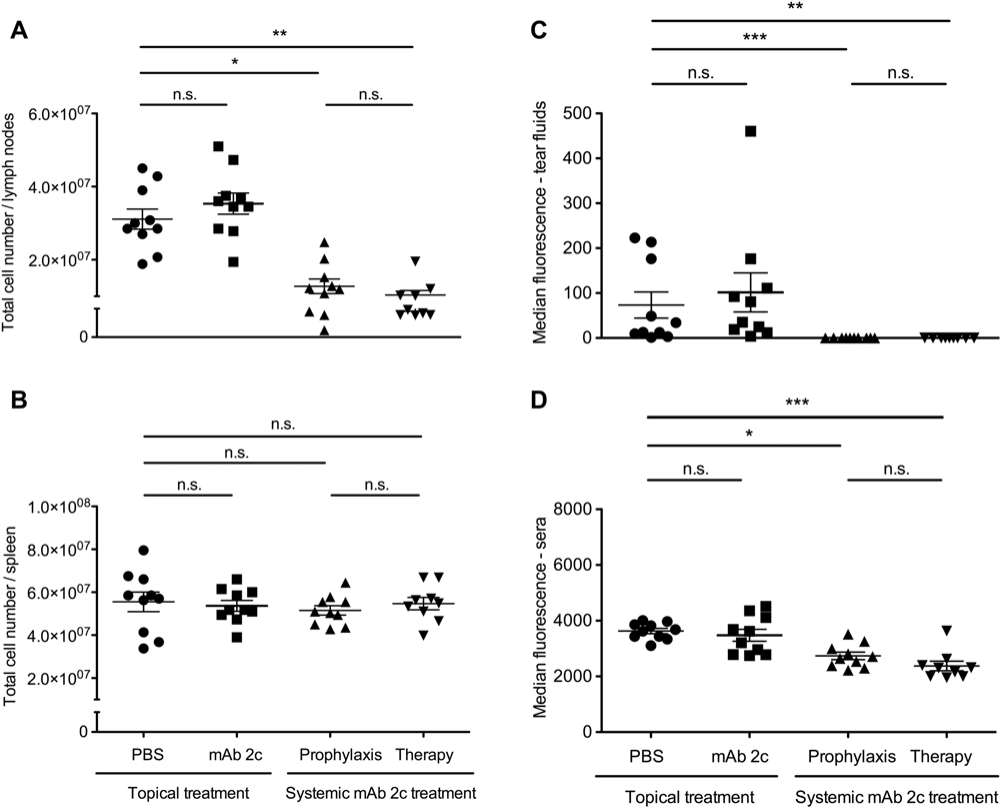
Influence of mAb 2c treatment on the cellular and humoral immune response to HSV-1 of corneally infected mice. The reduced virus titers correlate with reduced anti-HSV immune responses. The total numbers of cells in draining lymph nodes (DLN) **(A)** and spleens **(B)** and the relative antibody titers in 10 µl tear fluids **(C)** and 60 µl sera **(D)** of mice on day 14 post infection are shown. Each dot represents a single mouse. Differences between the groups were statistically significant by a nonparametric ANOVA one way test (*P < 0.05; **P < 0.01; ***P < 0.001). Error bars represent the SEM.

The significantly decreased inflammatory reaction and cell infiltration in the corneas of systemically treated mice lead to the assumption that mAb 2c may also influence the systemic immune response after HSV-1 infection. To further investigate this, we examined the total cell numbers in draining lymph nodes (DLN) and spleens, and the HSV-1 specific antibody titers in sera and tear fluids of mice on day 14 post infection. In accordance with the clinical observations, the DLNs of control mice or mice receiving the topical treatment were markedly enlarged. The total numbers of cells in the DLNs were 3.1 × 10^7^ ± 8.7 × 10^6^ (controls) or 3.5 × 10^7^ ± 9.2 × 10^6^ (topical treatment) respectively which was significantly higher when compared to systemically treated mice (prophylaxis: 1.3 × 10^7^ ± 1.0 × 10^6^; therapy: 1.0 × 10^7^ ± 3.7 × 10^6^) (Fig. 6A). This difference was not observed in the spleens of mice from different groups (Fig. 6B). Significantly lower antibody titers were found in sera and tear fluids of systemically treated mice when compared to the control or topical-treatment group (Fig. 6C and D). No HSV-specific antibodies were detectable in tear fluids of systemically treated mice. In contrast, similar antibody levels were found in the tear fluids of topically treated and control mice. Taken together, these results demonstrate that both the cellular and humoral immune response against HSV-1 was clearly decreased after systemic mAb 2c application.

### Reduced HSV-1 specific immune response in systemically treated mice

**Figure 7:**
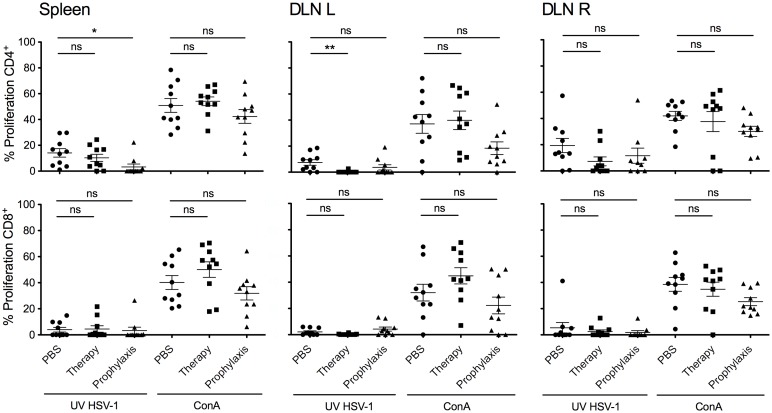
Proliferative response of CD4^+^ and CD8^+^ lymphocytes after mAb 2c application. Lymphocytes derived from spleens or DLNs (R = ipsilateral, L = contralateral) of HSV-1 KOS infected mice receiving prophylactical or postexposure treatment (therapy) with mAb 2c were stimulated either with UV‑inactivated HSV-1 or Concavalin A for four days. Proliferation of CD4^+^ and CD8^+^ lymphocytes was determined by flow cytometry. Differences between the groups were statistically significant as calculated by a nonparametric ANOVA one way test (*P < 0.05; **P < 0.01). Error bars represent the SEM.

**Figure 8:**
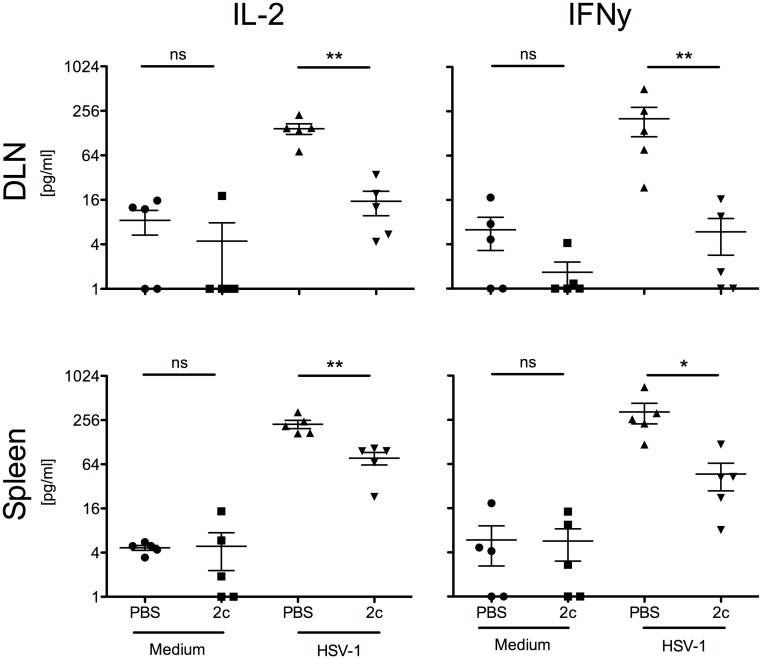
Reduced IL-2 and IFN-γ production by systemic mAb 2c application. BALB/c mice (n = 5) were intravenously injected with 300 µg mAb 2c before corneal infection with HSV-1 KOS (1x105 PFU/cornea). At day 14 post infection, spleens and draining lymph nodes were isolated and homogenized. Cells were cultivated in triplicates in the presence of 2 × 10^7^ PFU UV-inactivated HSV-1 or medium alone for stimulation. After 24 h, the contents of IL-2 and IFN-γ in cell supernatants were quantified by ELISA. Statistical analysis was undertaken with a nonparametric ANOVA test. Comparisons were considered significant at *P < 0.05. Error bars represent the standard error of the mean.

The decreased cell numbers in draining lymph nodes of mAb 2c treated mice indicated that the systemic route of application might reduce the HSV-1 specific cellular immune response. To specify the role of CD4^+^ and CD8^+^ lymphocytes after antibody therapy a subsequent analysis of T cell responses was performed. Proliferative responses of splenocytes or DLN-lymphocytes after stimulation with HSV-1 antigen revealed that the HSV-1 specific immune response was mainly driven by CD4^+^ T cells (Fig. 7). The proliferative CD4^+^ T cell response was markedly reduced when mice were systemically treated with mAb 2c (Fig. 7). CD4^+^ Type-1 T lymphocytes (Th1) predominantly secreting interleukin 2 (IL-2) and IFN-γ were shown to induce immunopathology in the course of corneal disease [36–38]. To investigate whether mAb 2c treatment influences the secretion of IFN-γ and IL-2, we pretreated 5 BALB/c mice with either 300 µg mAb 2c or PBS by intravenous injection 24 h before corneal HSV-1 KOS infection. After 14 days we analyzed the cytokine production of lymphocytes isolated from draining lymph nodes or spleens after stimulation with UV-inactivated HSV-1 KOS. The secretion of IFN-γ and IL-2 was significantly reduced in DLN-lymphocytes and splenocytes isolated from mice systemically treated with mAb 2c (Fig. 8). Taken together, these results confirm that mAb 2c significantly reduces HSV-1 specific CD4^+^ T cell responses in systemically treated mice.

### Effective neutralization of HSV-1 by mAb 2c at the site of infection

**Figure 9:**
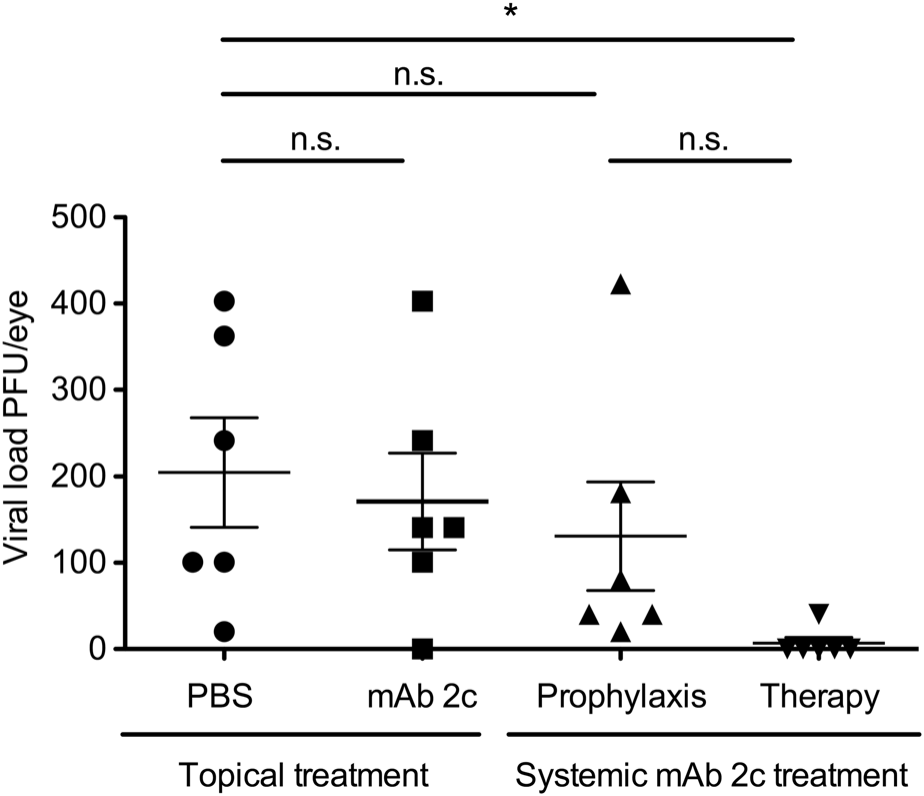
Effective virus neutralization in HSV-1 KOS infected eyes by mAb 2c. Representatively, six mice from each group were killed at day 5 post infection. Primary infected eyes were homogenized and inspected for viral loads using a standard plaque assay. Statistical analysis was undertaken with a nonparametric ANOVA test. Comparisons were considered significant at *P < 0.05. Error bars represent the SEM.

To investigate whether topical or systemic treatment with mAb 2c decreases viral loads at the site of infection, we isolated the initially infected eyes of 6 representative mice of each group on day 5 after infection and examined the virus content using a standard plaque assay. Systemically applied mAb 2c mediated substantial reduction of HSV-1 in infected eyes (Fig. 9). A considerable reduction of viral loads was observed in mice receiving a single dose of 300 µg mAb 2c 24 h before infection. Markedly, a significant reduction of viral loads was detected in mice that received three therapeutic doses (300 µg) of mAb 2c, starting 24 h after infection (Fig. 9). In this group the virus was completely eliminated in 5 out of 6 mice. In contrast, no reduction of virus titers was observed in mice treated with mAb 2c containing eye drops. These results clearly demonstrate that mAb 2c effectively reduces HSV-1 loads at the corneal site of infection when applied systemically. These results are consistent with the course of the disease and indicate that effective virus neutralization might be essential for the prevention of HSK.

### Effective reduction of virus spread to and reactivation from TG in mAb 2c treated mice

**Figure 10:**
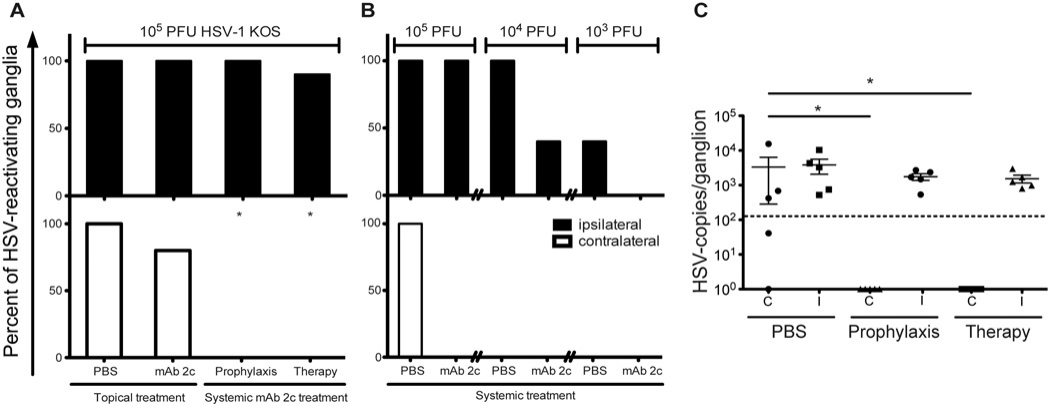
Reduced extent of HSV reactivations from trigeminal ganglia of mice systemically treated with mAb 2c. **(A)** Percentage of reactivating ipsilateral or contralateral trigeminal ganglia from HSV‑1 KOS infected mice is shown. Trigeminal ganglia were isolated on day 14 after infection and co-cultured with confluent Vero cells for three weeks to investigate the reactivation of virus. The occurrence of a cytopathic effect was taken as proof of virus reactivation. Each group consisted of ten mice. The differences in the total numbers of reactivating virus between the ipsilateral and contralateral ganglia were examined with Fisher´s exact test. Comparisons were considered significant at *P < 0.05. **(B)** Subsequent investigation of the impact of prophylactic mAb 2c application at 24 h before infection on the frequency of reactivating virus after infection with decreasing viral loads. Each group contained 5 mice. **(C)** Quantification of HSV-1 DNA in trigeminal ganglia isolated on day 14 after infection. Each group contained 5 mice. Statistical analysis was undertaken with a nonparametric ANOVA test. Comparisons were considered significant at *P < 0.05. Error bars represent the standard error of the mean. I = ipsilateral; C = contralateral.

The severity of corneal disease strongly correlates with the frequency of HSV-reactivations from latency in HSV-infected patients. Viewed in this context, we hypothesized that limiting the viral spread of HSV to the anatomically related neurons where the virus then usually establishes latency may have an advantageous effect on the occurrence of reactivations. Consequently, we investigated whether mAb 2c is capable of inhibiting the viral spread of HSV to the trigeminal ganglia by a virus reactivation assay. Therefore, trigeminal ganglia were isolated from the ipsilateral and contralateral site of infected eyes on day 14 after infection. The ganglia were co-cultured with Vero cells for three weeks, and monitored for viral reactivations. Recovery of HSV-1 was observed in all ipsilateral and contralateral TG isolated from mice of the control group (10/10). In topically treated mice, reactivated virus was similarly found in 10/10 of ipsilateral TG and in 8/10 of contralateral TG, revealing no significant difference when compared to the control group. In contrast, systemically applied mAb 2c significantly limited the frequency of virus reactivations from trigeminal ganglia. Although virus could be reactivated from 10 out of 10 (prophylaxis) or 9 out of 10 (therapy) ipsilateral ganglia, no virus reactivation was found at the contralateral site of the infection. These data indicate an effective inhibition of virus spread from the ipsilateral to the contralateral ganglia (Fig. 10A). To verify our findings we repeated this experiment (5 mice each group) under the same conditions and quantified HSV-1 DNA from trigeminal ganglia. No HSV-DNA could be detected in contralateral ganglia of systemically mAb 2c treated mice. In contrast, HSV-DNA was detected in 5/5 ipsilateral and 4/5 contralateral ganglia in the PBS control group (Fig. 10C).

Interestingly, the reactivation experiments revealed no difference between groups receiving mAb 2c prior or post infection. However, the transmission of virus to the TG of the infected eye could not be prevented in mice infected with a high dose (1 × 10^5^ PFU) of HSV-1 KOS. To investigate whether the viral spread to the ipsilateral TG may be completely blocked by mAb 2c in a low-dose HSV infection, 5 mice per group were infected with decreasing viral doses (10^5^, 10^4^ or 10^3^ PFU) of HSV-1 KOS 24 h after intravenous application of 300 µg mAb 2c. Reactivating virus from ipsilateral TG could be detected in 5/5 or 2/5 untreated mice infected with 10^4^ or 10^3^ PFU HSV-1 KOS. In contrast, the frequency of reactivating virus was markedly reduced in mice systemically treated with mAb 2c. Reactivating virus could be detected in 3/5 mice infected with 10^4^ PFU of HSV-1 KOS, and no reactivations were observed from ganglia of mice infected with 10^3^ PFU of HSV-1 KOS (Fig. 10B). These results demonstrate that the transmission of virus from the periphery to the trigeminal ganglia might be inhibited by mAb 2c in a virus infection dose-dependent manner.

### Prevention of HSK by the humanized antibody mAb hu2c

**Figure 11:**
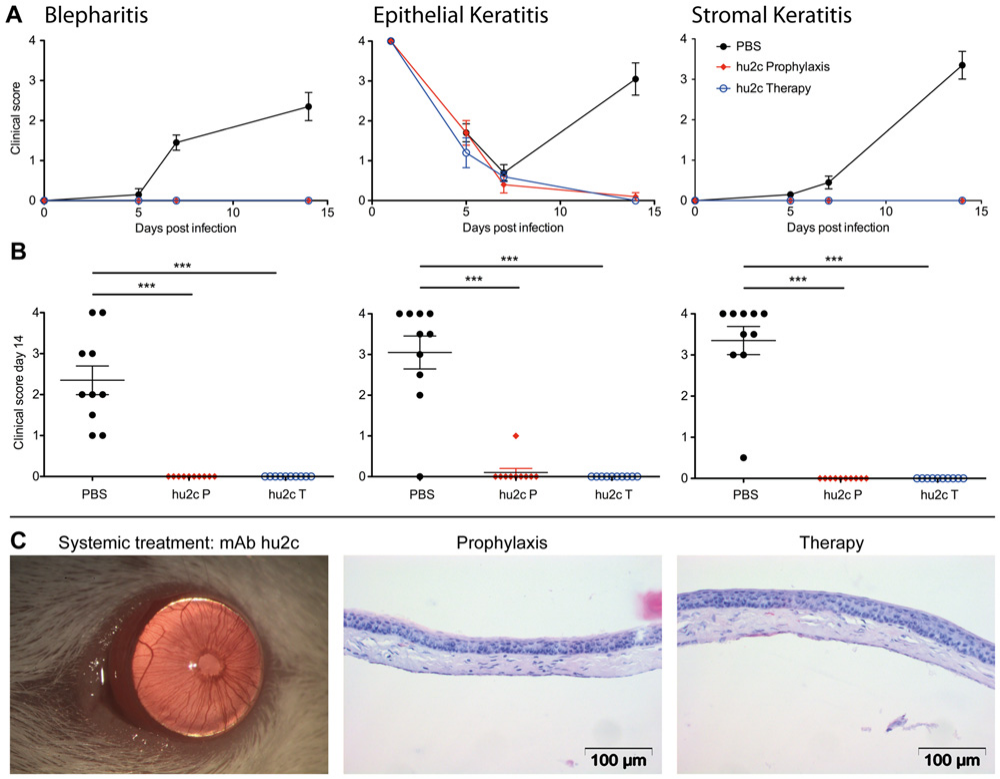
Prevention of corneal HSV-1 KOS infection in mice by the humanized antibody mAb hu2c. The clinical disease scores of infected mice for blepharitis, epithelial defects and corneal opacity (stromal keratitis) are shown as an average score of ten mice per group over a period of 14 days **(A)** or as dot plot **(B)** taken on day 14 after infection. Every dot represents a single mouse. Differences between the groups were statistically significant by a nonparametric ANOVA one way test (**P < 0.01; ***P < 0.001). Error bars represent the SEM. PBS-treated group showed in Fig. 3 serves as control. Pictures and histological stainings representative for mice systemically treated with mAb hu2c are shown **(C)**. Haematoxylin-eosin staining. Magnification: 120x. Scale bars:100 μm.

With regards to the future clinical application of the HSV-gB-specific monoclonal antibody in humans for the prevention of HSK, we subsequently investigated the protective effect of the humanized variant of this antibody (mAb hu2c) in corneally HSV-1 infected mice. Since the topical route of application showed no protective effect on the progress of corneal disease, only the systemic route of application was investigated for mAb hu2c. Mice were infected with HSV-1 KOS and systemically treated with mAb hu2c prior of post HSV-1 infection as described above for the parental mouse antibody. The humanized antibody proved to be equally effective in prevention of HSK when compared to the mouse antibody. The accompanying symptoms of HSK (blepharitis, epithelial defects and HSK) were significantly (P < 0.05) decreased in mice systemically treated with mAb hu2c, compared to controls (Fig. 11). All treated mice showed normal eyelids with almost no inflammation. The eye-blink reflex was intact and similar to uninfected corneas (data not shown). Taken together, both the parental mouse antibody as well as its humanized variant showed equal protective effect on the development of HSK and blindness in a highly relevant mouse model.

### Detection of systemically administered mAb hu2c at the infected cornea

**Figure 12:**
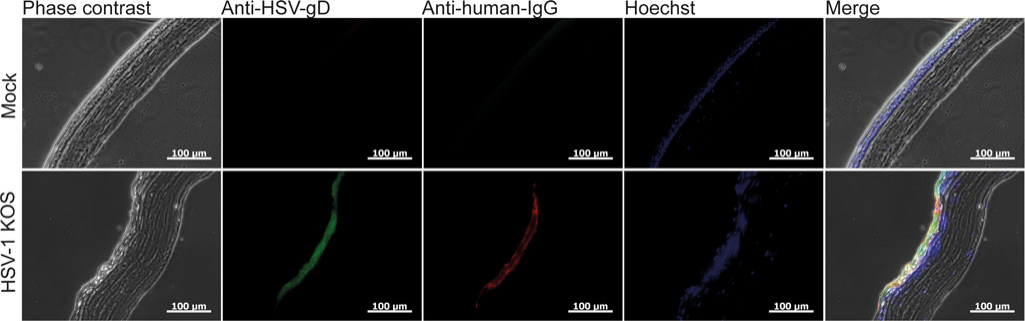
Colocalization of mAb hu2c and HSV antigens in HSV-1 KOS infected corneas. Mice were intravenously injected with 300µg of mAb hu2c (lower line) or PBS (upper line) at 48h after corneal infection with HSV-1 KOS. Six hours after injection, eyes were removed and sectioned. HSV-1 infection was stained with polyclonal goat anti-HSV-1 FITC conjugated antibodies and bound humanized antibody was detected with a Cy3-conjugated goat anti-human IgG secondary antibody. Nuclei were stained with Hoechst. Immunofluorescence images were acquired with a Zeiss Observer Z1 fluorescence microscope at a 200-fold magnification. Scale bars: 100 μm.

The absence of HSV-specific antibodies in tear fluids of systemically treated mice suggests that the presence of antibodies in tear fluids may have no impact on the disease progression. However, reduced viral loads in eyes of systemically treated mice indicated that the injected antibodies probably neutralize the virus. To clarify whether systemically applied mAb hu2c actually can reach the corneal tissue to exhibit antiviral activity, we examined corneal sections derived from HSV-1 KOS infected mice intravenously injected with mAb hu2c at 48h post infection by immunofluorescence. The eyes were isolated and sectioned six hours after injection in order to allow the antibody to distribute to the corneal tissue. The humanized antibody was detected in HSV-infected corneas when colocalized with HSV antigens. In contrast, no antibody could be detected in uninfected corneas, excluding unspecific binding or background fluorescence (Fig. 12). These results provide evidence for a successful distribution of mAb hu2c to the infected cornea.

## Discussion

Despite enormous medical progress over the last decades, Herpetic Stromal Keratitis still remains the leading cause of infectious corneal blindness. Until today, there is no effective vaccine available [39, 40]. The use of antiviral drugs is widespread, but limited due to toxicity and viral resistance [41]. The increasing prevalence of acyclovir and multidrug-resistant HSV-strains (6.4%) among patients with HSK is the major cause for therapy failure of inflammatory corneal HSV-infections [8, 42]. The use of monoclonal antibodies for the prevention of corneal HSV-infections was broadly discussed in previous studies [7, 43–47]. Monoclonal antibodies directed against HSV glycoproteins gB, gC, gD, and gE showed beneficial effects on the severity of corneal disease in mice in former studies [44]. Although limited protection from HSK could also be observed in mice treated with non-neutralizing monoclonal antibodies specific to HSV-1 gC, the best therapeutic effects could be achieved in mice treated with the neutralizing monoclonal antibodies Fd79 and mAb 8D2, specific for the glycoproteins gB and gD, respectively [7, 43, 44]. Nonetheless, antibody pretreatment was even not investigated (Fd79) or has failed (mAb 8D2) to prevent the development of corneal cloudiness [43]. To date, none of these antibodies was further reported for clinical use.

Consistent with the finding that several experimental HSV vaccines were protective in animal models but not in humans, recent studies indicate that the quality of humoral immune responses against HSV significantly differs between humans, guinea pigs and mice. While the recombinant gD vaccine induces strongly neutralizing antibodies in animal models, only weakly neutralizing responses were detectable in human vaccinees. The vast majority of the gD-specific human antibodies did not efficiently compete with gD epitopes recognized by highly neutralizing monoclonal antibodies derived from mice [48]. These results suggest that mice are capable of mounting a more potent antibody response against HSV than humans are. Against this background, the evaluation of a potent neutralizing mouse antibody for a future clinical use seems to represent a promising strategy to combat severe HSV infections.

Due to the persisting clinical need to overcome drug-resistant HSV-infections, we recently humanized the gB-specific monoclonal antibody mAb 2c that is capable of neutralizing ACV- and multi-resistant isolates from bone marrow transplanted patients in vitro and in the highly immunosuppressed NOD/SCID mouse model [11]. The humanized antibody mAb hu2c and the parental mouse antibody both recognize the same epitope that is highly conserved between HSV-1 and HSV-2 and essential for viral fitness. Mutations within both epitope subdomains resulted in a dramatic loss of infectivity [13]. Both antibodies exhibit equal neutralizing properties towards HSV-1 and HSV-2, independently from complement or antibody-dependent cellular effector functions [15].

In the present study we first examined the antiviral efficacy of the murine mAb 2c in the prevention of corneal HSV-infections in the well-established murine model of HSK. Subsequently we focused on the impact of antibody treatment on the level of immune response to HSV that is associated with the severity of corneal disease. With respect to the future clinical application, we finally carried out investigations whether the humanized variant mAb hu2c prevents HSK in a comparable manner to that of the parental mouse antibody.

Systemic antibody administration resulted in total protection of mice from HSV-1 KOS induced signs like blepharitis, epithelial defects or HSK after prophylaxis or post-exposure treatment. However, systemic antibody administration appears to be crucial for effective prevention of HSK, since repeated topical applications of mAb 2c were not effective. Other groups have also reported the failure of topical antibody treatment in the prevention of HSK [43]. The mechanism behind this finding remains controversial. It is generally believed that antibodies protect by neutralizing the extracellular virus, inhibiting the direct virus transmission between adjacent cells or by mediating the lysis of virus-infected cells [43]. To exert the neutralization of the virus, antibodies need to reach the infected corneal tissue. Systemically applied antibodies were found to enter the corneal tissue by diffusion of plasma IgG from the peripheral blood vessels and exhibit neutralizing activity [49, 50]. In contrast, topical administration of neutralizing antibodies is ineffective, most probably because IgG antibodies with a molecular weight of 149 kDa have a limited capacity to penetrate the normal cornea and thus fail to neutralize the virus [50, 51]. However, topical routes of administration are less invasive and probably better tolerated when compared to systemic application. Antibody Fab fragments (50 kDa) or single-chain fragments (26 kDa) lacking the constant-regions domains of whole antibodies are relatively small and able to penetrate the cornea [52]. Recently, a Fab antibody fragment (AC-8) specific to HSV glycoprotein D has been used topically to prevent HSK. Actually, treatment with AC-8 moderately reduced the ocular disease scores, but was not as effective as a standard therapy with trifluorthymidine [41]. Nevertheless, Fab fragments derived from mAb 2c IgG are almost ineffective in virus neutralization, since bivalency is essential for its proper function [11]. Hence, subsequent studies with recombinant mAb 2c derivates are clearly needed to develop appropriate antibody constructs for topical application.

HSK has been shown to have an inflammatory immunopathological origin [53]. The exact mechanism by which monoclonal antibodies specific for HSV glycoproteins exert protective effects on the development of HSK remains unknown. Several observations indicate that neutralizing antibodies prevent corneal disease more effectively when compared to non-neutralizing antibodies [7]. On the contrary, a neutralizing antibody specific for the glycoprotein D that effectively prevented HSK had no significant impact on the virus load in the eye [43]. The antibody investigated in this study effectively neutralized HSV-1 in the eye. These results suggest that virus neutralization actually is not the only mechanism involved in the prevention of corneal disease. However, previous studies have shown that HSV-specific antibodies may protect against HSK by inhibiting the production of chemokines believed to promote an inflammatory immune response to HSV [45]. In accordance with these findings, the limitation of cell infiltration to the cornea, the reduction of total cell numbers in the draining lymph nodes, the reduced CD4^+^ T cell response and the significantly reduced antibody levels in tear fluids and sera of systemically treated mice indicate that mAb 2c may also protect from HSK by a significant reduction of the immune response. Most probably, the reduced immune response is a consequence of virus neutralization by mAb 2c. Notably, T-cells responding to HSV-1 infection play a critical role in the initiation of tissue damage within the cornea. Neutrophiles and monocytes also infiltrate the cornea and are involved in pathogenicity [30, 45]. There is substantial evidence that the Type-1 T lymphocytes (Th1) predominantly secreting interleukin 2 (IL-2) and interferon (IFN)-γ are pathogenic in the evolution of corneal disease, since the neutralization of IL-2 and IFN-γ results in the remission of HSK [36–38, 54]. In contrast, Th2 associated cytokines IL-4 and IL-10 suppress the development of HSK [22].

It is commonly accepted that the development and progression of HSK strongly depends on the frequency of HSV-reactivations [55, 56]. Antibodies inhibiting neuronal spread via cell-to-cell transmission may significantly reduce the frequency of reactivations and thus have a sustainable impact on the emergence of HSK [56]. Although the exact mechanism of viral DNA transport or release from the axons is still unknown, several observations also suggest that antibodies could directly interfere with the axonal HSV-1 spread in vitro and in vivo **[57, 58]. In the present study we demonstrated that mAb 2c and its humanized counterpart both are capable to interfere with the cell-to-cell transmission between epithelial cells and neurons. Moreover, we found that systemic mAb 2c treatment markedly limited the establishment of latency to the ipsilateral trigeminal ganglion, indicating that neuronal spread along neuronal fibers to the contralateral ganglion was interrupted.

Finally, systemic treatment with the humanized antibody mAb hu2c mediated equal protection from the development of HSK when compared to the murine antibody. This correlates with the results from a former study in which both antibodies showed equal antiviral activity under immunosuppressed conditions [11, 15].

In conclusion, we have shown that the neutralizing monoclonal antibody mAb 2c effectively neutralizes ACV resistant isolates from patients with frequently recurring corneal infections in vitro, and prevents the development of HSK in mice. Moreover, the humanized variant mAb hu2c prevented the development of HSK with the same efficacy when compared to the parental antibody. These features warrant the clinical development of this antibody for treatment and prevention of severe, drug-resistant corneal infections in patients.
